# Impact of Training and Municipal Support on Primary Health Care–Based Measurement of Alcohol Consumption in Three Latin American Countries: 5-Month Outcome Results of the Quasi-experimental Randomized SCALA Trial

**DOI:** 10.1007/s11606-020-06503-9

**Published:** 2021-01-19

**Authors:** Peter Anderson, Jakob Manthey, Eva Jané Llopis, Guillermina Natera Rey, Ines V. Bustamante, Marina Piazza, Perla Sonia Medina Aguilar, Juliana Mejía-Trujillo, Augusto Pérez-Gómez, Gill Rowlands, Hugo Lopez-Pelayo, Liesbeth Mercken, Dasa Kokole, Amy O’Donnell, Adriana Solovei, Eileen Kaner, Bernd Schulte, Hein de Vries, Christiane Schmidt, Antoni Gual, Jürgen Rehm

**Affiliations:** 1 Department of Health Promotion, CAPHRI Care and Public Health Research Institute, Maastricht University, Maastricht, The Netherlands; 2 Population Health Sciences Institute, Newcastle University, Newcastle upon Tyne, UK; 3 Institute for Clinical Psychology and Psychotherapy, TU Dresden, Dresden, Germany; 4 Center for Interdisciplinary Addiction Research (ZIS), Department of Psychiatry and Psychotherapy, University Medical Center Hamburg-Eppendorf, Hamburg, Germany; 5 Univ. Ramon Llull, ESADE, Barcelona, Spain; 6 Institute for Mental Health Policy Research, CAMH, Toronto, ON Canada; 7 Instituto Nacional de Psiquiatría Ramón de la Fuente Muñiz, Ciudad de México, CDMX Mexico; 8 School of Public Health and Administration, Universidad Peruana Cayetano Heredia, Lima, Peru; 9 Corporación Nuevos Rumbos, Bogotá, Colombia; 10 Addictions Unit, Psychiatry Dept., Hospital Clínic, Barcelona, Spain; 11 Red de Trastornos Adictivos, Instituto Carlos III, Madrid, Spain; 12 Institut d’Investigacions Biomèdiques August Pi Sunyer (IDIBAPS), Barcelona, Spain; 13 Dalla Lana School of Public Health, University of Toronto, Toronto, ON Canada; 14 Department of Psychiatry, University of Toronto, Toronto, ON Canada; 15 Department of International Health Projects, Institute for Leadership and Health Management, I.M. Sechenov First Moscow State Medical University, Moscow, Russian Federation

**Keywords:** primary health care, municipal action, heavy drinking, Institute for Health Care Improvement, implementation, measurement of alcohol consumption, AUDIT-C, brief advice, Colombia, Peru, Mexico

## Abstract

**Purpose:**

We aimed to test the effects of providing municipal support and training to primary health care providers compared to both training alone and to care as usual on the proportion of adult patients having their alcohol consumption measured.

**Methods:**

We undertook a quasi-experimental study reporting on a 5-month implementation period in 58 primary health care centres from municipal areas within Bogotá (Colombia), Mexico City (Mexico), and Lima (Peru). Within the municipal areas, units were randomized to four arms: (1) care as usual (control); (2) training alone; (3) training and municipal support, designed specifically for the study, using a less intensive clinical and training package; and (4) training and municipal support, designed specifically for the study, using a more intense clinical and training package. The primary outcome was the cumulative proportion of consulting adult patients out of the population registered within the centre whose alcohol consumption was measured (coverage).

**Results:**

The combination of municipal support and training did not result in higher coverage than training alone (incidence rate ratio (IRR) = 1.0, 95% CI = 0.6 to 0.8). Training alone resulted in higher coverage than no training (IRR = 9.8, 95% CI = 4.1 to 24.7). Coverage did not differ by intensity of the clinical and training package (coefficient = 0.8, 95% CI 0.4 to 1.5).

**Conclusions:**

Training of providers is key to increasing coverage of alcohol measurement amongst primary health care patients. Although municipal support provided no added value, it is too early to conclude this finding, since full implementation was shortened due to COVID-19 restrictions.

**Trial Registration:**

Clinical Trials.gov ID: NCT03524599; Registered 15 May 2018; https://clinicaltrials.gov/ct2/show/NCT03524599

**Supplementary Information:**

The online version contains supplementary material available at 10.1007/s11606-020-06503-9.

## INTRODUCTION

Alcohol use is a leading risk factor for ill-health and premature death, increasing a wide range of cancers, and cardiovascular and gastrointestinal diseases^1–4^. Within the World Health Organization’s (WHO) SAFER initiative, facilitating population-level health service access to measurement of alcohol consumption, and delivering brief advice and treatment as required, is one of five high-impact strategies to reduce the harm done by alcohol^5^.

A number of meta-analyses and systematic reviews have demonstrated the impact^6–8^ and cost-effectiveness^9, 10^ of primary health care (PHC)–based measurement and brief advice programmes in reducing heavy drinking.

Despite the evidence of impact, few governments seeming willing to undertake the necessary investments to ensure countrywide implementation, and the global penetration of such programmes remains generally very low^10–16^. While no published data is available of the extent of PHC-based measurement and brief advice activity in reducing heavy drinking in the three study countries of the SCALA trial (Colombia, Mexico, and Peru), the country investigators report that such work is not normally undertaken.

Lack of motivation, being too busy, and lack of adequate training and support materials are expressed as important barriers to the delivery of measurement and brief advice programmes^17, 18^. Conversely, having more time, having less intense programmes to deliver, and having more active patients asking for alcohol advice have been expressed as facilitators for implementing measurement and brief advice^19, 20^.

Within the field of implementation science^21^, systematic reviews^22, 23^ and multi-country studies^24–27^ have demonstrated the importance of providing training to PHC providers in increasing their activity in measuring alcohol consumption and giving brief advice to identified heavy drinkers to help reduce their alcohol consumption. It has been widely proposed that PHC-based activities within complex health systems could be improved by addressing underlying structural and support factors^28^, embedding PHC-based measurement and brief advice programmes within the frame of supportive community and municipal environments^29–33^.

The international SCALA project (Scale-up of Prevention and Management of Alcohol Use Disorders in Latin America, www.scalaproject.eu) aims to evaluate the impact of multilevel interventions on rates of health care–based measurement, advice and treatment for heavy drinking of alcohol and comorbid depression^34, 35^ (although, in this paper, we only report results for alcohol).

Latin American countries are chosen for three reasons: the regional importance of alcohol as a risk factor for morbidity and premature death^36^; health system reforms that emphasize primary health care as a vehicle to achieve universal health coverage and prevention^37–39^; and fast track research from high-income countries to Latin American middle-income countries^40–46^.

We test the following three pre-published hypotheses^35^:

- **Hypothesis 1:** For PHC centres whose providers have received training, the presence of municipal support, designed and implemented for the purpose of the SCALA study, leads to more sustainable coverage of alcohol measurements than the absence of municipal support;
- **Hypothesis 2**: In the absence of municipal support, PHC centres whose providers receive training obtain higher coverage than PHC centres whose providers have not received training; and
- **Hypotheses 3**: In the presence of municipal support, use of a less intense version of a delivered clinical package and training does not lead to less coverage of alcohol measurements than delivery of a standard more intense clinical package and training.

There are deviations to the protocol^35^, due to COVID-19 illness. Our original plan was for an 18-month implementation period with formal evaluation points at 6, 12, and 18 months. According to the WHO COVID-19 dashboard^47^, Colombia, Mexico, and Peru have been amongst some of the worst affected countries in the world for COVID-19. Due to the social distancing and ‘lock-down’ measures implemented in the countries as a mitigation response to COVID-19 illness, much routine preventive work was halted and many providers were confined to home. This compromised continued measurement and advice activities as part of SCALA, and an inability for the local researchers to visit the PHC centres. Thus, we had to pause the work and we chose to bring forward the first 6-month evaluation time point to 5 months, given restrictions on preventive work beyond COVID-19 and on the uncertainty as to when and how full implementation might restart.

## METHODS

**Figure 1:**
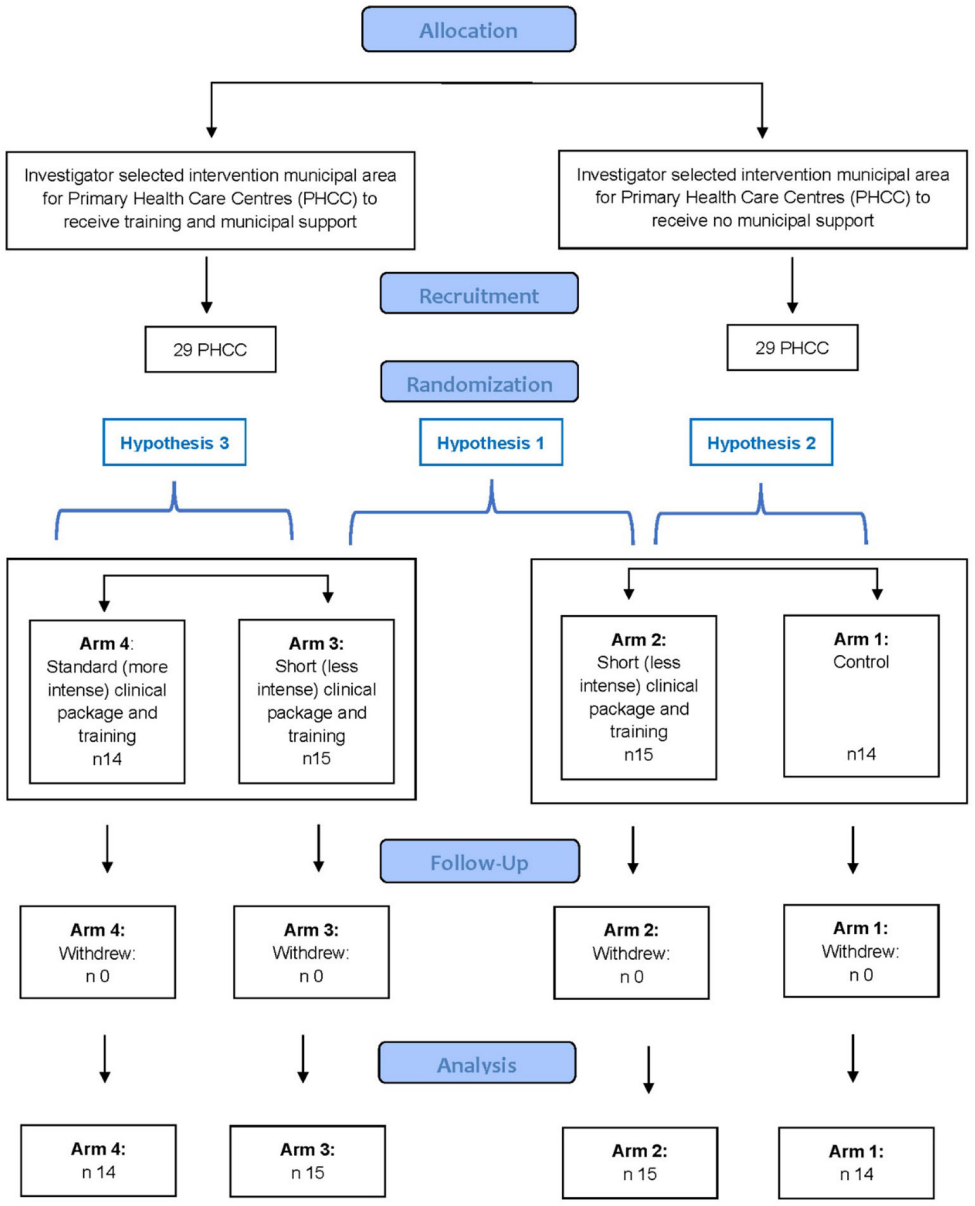
Study flow chart.

The study is a quasi-experimental design,^48^ comparing changes in measurement of alcohol consumption between PHC centres in intervention municipal areas and PHC centres in similar control municipal areas (Fig. 1).

One intervention municipal area was investigator-selected from each of Bogotá (Colombia), Mexico City (Mexico), and Callao–Lima (Peru). One control municipal area was investigator-selected from each of the same cities, on the basis of comparability in terms of socio-economic characteristics, and with sufficient geographical separation to minimize spillover effects from the intervention municipal area. Randomized selection of the municipal areas was not feasible due to organizational limitations, and the need to obtain approval from municipal authorities.

Within the municipal areas, the units of allocation and analysis, i.e., study participants, are PHC centres and the providers working in them. Centres were invited to join the study until a minimum of 27 were achieved within each of the two municipal areas (intervention and control) across the three countries (nine per municipal area within each of the three countries). Within each centre, eligible providers include any fully trained health care provider, with written informed consent for participation. In the end, 58 centres were recruited, 29 in the intervention municipal area and 29 in the control municipal area.

Within the control municipal area, 14 centres were randomly allocated to control (arm 1), and 15 to receive less intense training to implement a less intense clinical package (arm 2). Within the intervention municipal area, in which municipal support was provided, 15 centres were randomly allocated to receive less intense training to implement a less intense clinical package (arm 3), and 14 were allocated to receive standard (more intense) training to implement a standard (more intense) clinical package (arm 4). Random allocation was stratified by country and undertaken using Excel random number generator.

The clinical package, tailored for local use, comprised measurement instruments, patient information and advice material, and provider guidance material, with the differences between the standard (more intense) and less intense clinical materials described in the published protocol^35^. The less intense version was a simplified version of standard UK-based materials^49^, deliverable in a reduced period of time, noting evidence that the duration of brief advice has little impact on outcome^7, 50, 51^.

All providers in arms 2–4 were given one and a half to 2 h tailored training in arms 2 and 3, and three and a half to 4 h tailored training in arm 4, after the baseline measurement period and before the implementation period. Training focussed on practical skills in undertaking alcohol measurement and in delivering brief advice, and on using the measurement instruments. The difference between arm 4 and arms 2 and 3 was to test hypothesis 3 (less intense clinical intervention and training does not lead to less coverage of alcohol measurements than the use of standard more intense clinical intervention and training). The training methods were the same across all three arms, using a modelling strategy based on videos, and tailored to the PHC settings and systems in the three Latin American countries.

In all arms, PHC providers were asked to measure the alcohol consumption of all adult patients who consulted for any reason using AUDIT-C^52^, with a stem question to exclude patients who had already completed the AUDIT-C during the study period. The three AUDIT-C questions were included in a paper tally sheet completed by the provider, in which the provider documented the outcome of the consultation (advice given, patient referred etc.).

The municipal support inputs to arms 3 and 4, based on the Institute for Healthcare Improvement’s frame for going to scale^28^, were designed, tailored, and implemented for the purposes of the SCALA study. They included five common components across the three countries^35^:

i. Creation of local stakeholder groups to advise on tailoring, support implementation, and review drivers of successful action;
ii. Appointment of local project champions to advocate for successful implementation;
iii. Implementation of five evidence-based adoption mechanisms;
iv. Implementation of five evidence-based support systems; and
v. Implementation of community-based communication campaigns.

### Data collection

Data were collected between April 2019 and April 2020. Before the start of baseline data collection, we obtained characteristics of the participating PHC centres, including the number of providers working in the centre by profession and the number of adults registered with the centre (Appendix Table 1). During the course of the study, providers completed the number of adult consultations on a monthly basis. During the baseline and the five implementation months, providers returned completed tally sheets, enabling a calculation of the numbers of adult patients whose alcohol consumption was measured.

### Definition of variables for analyses

All analyses were conducted at the PHC centre level. The primary outcome was the coverage of alcohol measurement, defined as the cumulative proportion of the adult (aged 18+ years) population registered with the PHC centre that has their alcohol consumption measured. The denominator definitions of registered population by country are summarized in Appendix Table 1. The nominator of the primary outcome was calculated by summing up the number of completed measurement instruments (AUDIT-C) per PHC Centre and month, before computing a cumulative variable of alcohol measurements across all months per PHC centre and month. The nominator measures the number of individual patients whose alcohol consumption was measured, rather than patient encounters. The final primary outcome variable is expressed as the cumulative number of alcohol measurements per 1000 registered patients. Two adjustment variables were considered: (1) dummy-coded country variables, to account for differences between countries; and (2) baseline coverage, to account for variations in measurement activity before training and municipal support were implemented. All descriptive and inferential analyses of the primary outcome were weighted for PHC Centre size, i.e., for the number of adults registered with the Centre. The weight variable was normalized by dividing the Centre size by the mean Centre size across all Centres.

### Power calculations

As detailed in the protocol^35^, amongst centres that did not receive municipal support, the planned study was powered to detect a doubling of cumulative coverage after 12 months from 3.25% of the registered adult population in control centres to 7.5% in centres whose providers had received training (82% power at a significance level of 5%). In the presence of training, the study was powered to detect a further doubling of cumulative coverage after 12 months from 7.5% in centres that did not receive municipal support to 15% in centres that did receive municipal support (96.5% power at a significance level of 5%)^53^.

### Statistical analyses

The distribution of the primary outcome is best described as negative binomial, Appendix Figure 1. For testing the three hypotheses, the primary outcome was analysed cross-sectionally using cumulative data at month 5, with negative binomial regressions comparing the dependent variable by exposure variable, while accounting for country differences and baseline measurement activity. Interaction effects of country and exposure were not significant, Appendix Table 2.

We report exponentiated coefficients of negative binomial regression analyses (with 95% confidence intervals), such coefficients being incidence rate ratios (IRR). The IRR is the ratio of the outcome (cumulative coverage) for the exposure happening (e.g., training) to the exposure not happening (e.g., no training).

All Peruvian PHC Centres had their fifth month of data collection completed by early February 2020. In Colombia and Mexico, several Centres would have completed their fifth month of data collection only post data closure. For these Centres, the last reported cumulative coverage rate was carried forward, assuming no further measurements, with the estimated impact of missing data being of negligible extent (Appendix Table 3).

Analyses were run with R version 3.6.1.^54^

## RESULTS

The municipal areas were similar in basic demographic variables (Appendix Table 4). At baseline, 58 PHC Centres (Colombia: 20; Mexico: 18; Peru: 20) participated in the study. In total, 622 providers (Colombia: 128; Mexico: 256; Peru: 238) consented to participate; 524 were recruited at baseline and 98 joined the study throughout the implementation period (Table 1).

**Table 1:** Sample Characteristics

	Arm 1 (*n* = 14)	Arm 2 (*n* = 15)	Arm 3 (*n* = 15)	Arm 4 (*n* = 14)
Total number of participating providers^a b^	126	206	173	144
Mean number of providers per Centre^a^ (SD^c^)	7.5 (7.4)	10.3 (6.3)	8.5 (5.9)	8.2 (5.7)
Mean number of registered patients per Centre (SD^c^)	23,967.3 (44,346.9)	11,164.0 (9936.4)	11,616.7 (7674.5)	14,020.4 (7961.5)
Mean number of consultations per provider per month (SD)	181.6 (126.7)	179.9 (132.8)	203.8 (134.7)	198.5 (150.4)

^a^Summed/averaged across baseline and 5-month implementation periods

^b^Some providers in Colombia worked in more than one Centre/arm; thus, the total across all arms is greater than the total number of providers participating in this study

^c^Standard deviation

No Centres dropped out during the study. The median number of registered patients per Centre at baseline was 9048 (mean = 15,006, SD = 23,555), with the largest Centre having more than five times more patients registered relative to the second largest (177,953 vs 31,501). After removing this outlier from this comparison, the registered patient population size did not differ by arm (ANOVA: F = 0.528, *p* = 0.655). Across the observed study period, the median number of participating providers per Centre was 7 (mean = 8.7, SD = 6.4), not differing by arm (ANOVA: F = 2.69, *p* = 0.056), and the median proportion of all providers within a Centre that participated in the study was 35.7% (mean = 40.4%, SD = 28.3%), not differing by arm (ANOVA treating the participation rate as continuous, normally distributed variable: F = 0.829, *p* = 0.484). The median number of monthly consultations per Centre (averaged over participating providers) was 132 (mean = 191.0, SD = 131.8), not differing by arm (ANOVA: F = 1.523, *p* = 0.219). The characteristics of the providers by study arm are summarized in Appendix Table 5.

### Training coverage

The proportion of providers across arms attending at least one training session was 72.3%, being higher in arms 2 and 4 (74.1% and 76.9% respectively) than in arm 3 (66.3%) (Appendix Table 5). Any heterogeneity across countries is accounted for by including country as dummy variable in the model.

### Municipal action

In each country, two or three community advisory board meetings of local stakeholders took place and one or two project champions provided ongoing implementation support (Appendix Table 6). Five adoption mechanisms and five support systems were wholly or partly implemented. A communication campaign was initiated in Mexico and Peru during month 4 of implementation and planned for month 6 in Colombia. Due to restrictions of COVID-19, the communication campaign was partially paused or delayed in all three intervention communities. Any heterogeneity across countries is accounted for by including country as dummy variable in the model.

### Coverage of alcohol measurements at baseline and month 5 across all arms

Upon completion of the 1-month baseline assessment, 2.1 per 1000 registered patients had their alcohol consumption measured (SD: 5.4). This baseline level is probably a consequence of all providers being asked to measure the alcohol consumption of all consulting adult patients, rather than an indicator of routine activity, which, prior to SCALA, was considered non-existent by the country investigators. The cumulative number rose to 11.7 (SD: 22.7) per 1000 after completing the 5-month implementation period.

### Evaluation of overall effects

**Figure 2:**
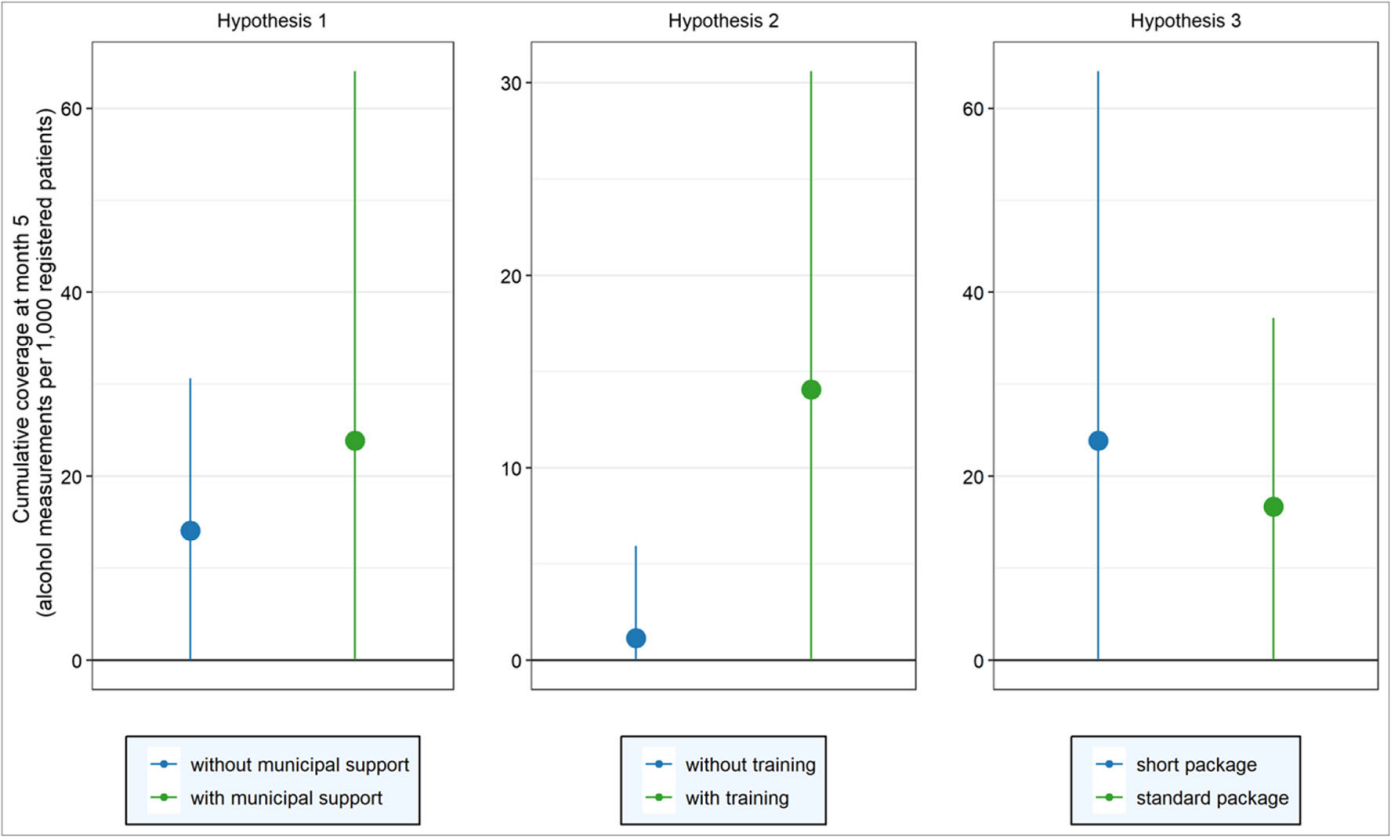
Average cumulative coverage at month 5 for each exposure group, as defined by each hypothesis (points indicate mean and vertical lines indicate mean ± one standard deviation).

The distribution of the cumulative coverage at month 5 by hypothesis is summarized in Figure 2 and described for each hypothesis. In Table 2, results of regression analyses testing the three hypotheses are presented. In this table, the coefficient of the exposure variable indicates the incidence rate ratios in measurement rates between the arms as postulated in the respective hypotheses. For example, an incident rate ratio of 9.8 in hypothesis 2 implies that, keeping the covariates constant, PHC centres in arm 2 (providers assigned to be trained), had, on average, a 9.8 times higher alcohol measurement coverage rate than PHC Centres in arm 1 (providers not assigned to be trained). The results are presented for each hypothesis below.

**Table 2:** Results of Regression Analyses for Evaluating Hypotheses 1–3

	Hypothesis 1	Hypothesis 2	Hypothesis 3
Exposure^a^	1.0 (0.6, 1.8)	9.8** (4.1, 24.7)	0.8 (0.4, 1.5)
Country (base: Colombia)			
Mexico	0.3** (0.1, 0.6)	0.4 (0.05, 2.0)	0.7 (0.3, 1.7)
Peru	0.2** (0.1, 0.5)	0.2* (0.04, 0.8)	0.3** (0.1, 0.7)
Baseline coverage rate	1.05* (1.0, 1.1)	1.08 (0.96, 1.4)	1.07** (1.0, 1.2)
Intercept	42.6** (17.5, 123.8)	3.7 (1.0, 21.0)	23.032** (9.2, 62.6)
Observations	30	29	29
Log Likelihood	− 80.8	− 70.5	− 90.1
Theta	2.6** (0.87)	0.9* (0.43)	1.8** (0.57)
Akaike Inf. Crit.	171.5	151.0	190.2

Presented are exponentiated coefficients of negative binomial regression analyses, which should be interpreted as incidence rate ratios. Numbers in brackets denote 95% confidence intervals

^a^Exposure variable defined by hypothesis: H1: without (base) vs with municipal support; H2: without (base) vs with training; H3: short (base) vs standard package

*p < 0.1; **p < 0.05; ***p < 0.01

#### Hypothesis 1: Increased Coverage Through Municipal Support (Arm 3 vs Arm 2)

Hypothesis 1 is comparing centres between two investigator-assigned municipal areas. Although the raw cumulative coverage at month 5 was higher in Centres receiving both municipal support and training (mean: 23.9 per 1000, SD: 40.1) relative to Centres receiving training only (mean: 14.1 per 1000, SD: 16.5) (Fig. 2), the difference was not significant (coefficient, incident rate ratio (IRR) = 1.0, 95% CI = 0.6 to 1.8, Table 2). Thus, hypothesis 1 is not confirmed.

#### Hypothesis 2: Increased Coverage Through Training (Arm 2 vs Arm 1)

Hypothesis 2 is comparing centres randomly allocated to separate arms. The raw cumulative coverage at month 5 was higher in PHCUs receiving training (mean: 14.1 per 1000, SD: 16.5) relative to PHCUs not receiving training (1.1 per 1000, SD: 4.8) (Fig. 2), and this difference was significant (coefficient, IRR = 9.8, 95% CI = 4.1 to 24.7) (Table 2), that is a 9.8-fold increase in measurement coverage amongst Centres receiving training. Thus, hypothesis 2 is confirmed.

#### Hypothesis 3: No Increased Coverage Through Longer Package (Arm 4 vs Arm 3)

Hypothesis 3 is comparing centres randomly allocated to separate arms. Although the raw cumulative coverage at month 5 was higher in Centres working with the less intense package (mean: 23.9 per 1000; SD: 16.7) relative to Centres working with the standard more intense package (16.7 per 1000; SD: 20.5) (Fig. 2), this difference was not significant (coefficient, IRR = 0.8, 95% CI = 0.4 to 1.5) (Table 2). Thus, hypothesis 3 is confirmed.

## DISCUSSION

The SCALA project was set up in middle-income countries to test the following: does training of providers, in the absence of municipal support, lead to a higher cumulative proportion of patients having their alcohol consumption measured (coverage), and, in the presence of training, does the extra provision of municipal-based support lead to improved coverage. We chose adult patient ‘coverage’ as our outcome measure, since this is similar to the blood pressure model, in which one is interested in the proportion of patients who have had their blood pressure measured^55, 56^. Measurement coverage is based on an assumption that advice to cut down is routinely given patients with a high measured alcohol consumption, which, in the ODHIN study, was just under 80%^26^.

As with the two previous international studies^24–27^, we demonstrated a clear impact of training (hypothesis 2, tested between centres randomly allocated to two separate arms). In the absence of municipal support, centres whose providers received training measured the alcohol consumption of a nearly ten times higher proportion of registered patients (coverage) than centres whose providers had not received training, although, as with the previous studies^24–27^, coverage remained small (14/1000 registered population).

Based on the conclusions of the WHO Phase IV study^29^, we had anticipated that municipal support would lead to higher coverage (hypothesis 1, tested between centres between from each of two investigator-assigned municipal areas), but we have not been able to demonstrate this. Municipal support is an action over time that might lead to cumulative effects over time. Furthermore, not all the municipal support interventions have been implemented as planned, in particular the community-based communication campaigns, due to lockdown restrictions following COVID-19. For these reasons, we think it premature to conclude that municipal support does not lead to higher coverage.

SCALA is an implementation action study utilizing an evidence-based clinical measurement and advice package. We considered it important to test whether or not coverage was dependent on the clinical package used (standard or less intense). As non-superiority is given (hypothesis 3, tested between centres randomly allocated to two separate arms), a shorter less intense package can be implemented as the norm.

### Strengths and Weaknesses

Our study has a number of strengths. SCALA is the first multi-country primary health care–based study on alcohol that we know of being implemented in middle-income countries. We use coverage of an adult practice population as the primary outcome measure^57^. We based our implementation model on that of the Institute for Health Care Improvement going to scale^28^, and we tailored our materials based on the tailoring for chronic diseases initiative^58^. We have built in mechanisms for replicability and options for exploitation for scale up^35^.

Our study, though, has a number of weaknesses. For one of our hypothesis, the impact of municipal support, we were not able to allocate randomly the two municipal areas. Nevertheless, the two areas were similar in core socio-demographic variables, and the lack of randomization of the areas is unlikely to be the cause of the rejection of the hypothesis. Social distancing measures and lock-down to mitigate the spread of COVID-19 illness meant that we have not been able to complete the full implementation of municipal support. On the other hand, for two of our hypotheses (2 and 3), both of which were confirmed, we were able to allocate randomly the centres between the arms.

While we cannot preclude the possibility of substantially different results based on 6 months of data, testing the hypotheses at an earlier point can be considered more conservative as effects would be expected to increase over time.

We have a number of plans to reset and restart the implementation once lock-downs and social distancing measures are relaxed. We will review and refocus the elements of municipal support, telescoping a sustained and intense action within a short period of time within a second 6-month implementation phase. We will continue to test our original first two hypotheses, but adding additional hypotheses to study the impact of COVID-19 mitigation measures, by comparing results between the first phase (reported here) and the second phase to be implemented. We are also developing tele-medicine approaches^59^ for delivering measurement and brief advice for heavy drinking, which we will separately evaluate.

### Implications

There are a number of practice and policy implications. We propose the use of coverage as the main outcome measure for similar implementation studies. Tailoring indicated the importance of keeping clinical and training packages as short and as simple as possible to enable widespread deployment. To increase coverage of alcohol measurement (and subsequent advice to identified heavy drinkers), health care providers require tailored skills–based training. This can be of relative short duration (2 h). Although we did not find added value of municipal support, we consider it premature to dismiss the need for municipal support, as disruption due to social distancing measures and lock-downs to mitigate spread of COVID-19 illness has not allowed us to test the full sustainable impact of municipal support as planned.

To deliver widespread implementation beyond the test phase, ministries of health at municipal and country levels are represented in the Community Advisory Boards created in each intervention municipality to ensure sustainability and to facilitate scale-up at municipal and country levels.^35^ SCALA works closely with the Pan American Health Organization (PAHO), with the principal investigator from Mexico being a Collaborating Centre with PAHO, to facilitate scale-up at Latin American levels.

## Supplementary Information

ESM 1 (DOCX 99 kb)
