# Supplementary material for: Impact of Training and Municipal Support on Primary Health Care–Based Measurement of Alcohol Consumption in Three Latin American Countries: 5-Month Outcome Results of the Quasi-experimental Randomized SCALA Trial
Source: J Gen Intern Med. 2021 Jan 19;36(9):2663–71. doi: 10.1007/s11606-020-06503-9 (PMC7815287; doi:10.1007/s11606-020-06503-9)
Supplement: Supplementary file 1 — (DOCX 99 kb) [file 11606_2020_6503_MOESM1_ESM.docx]

# Appendix

**Appendix Table 1** Denominator definitions by country

| **Colombia** |
| --- |
| The registered population corresponds to official reports received from directors of the hospitals, and covers people who are eligible to receive care at any PHC Centre belonging to the hospital. |
| **Mexico** |
| The PHC Centre population is provided by the statistical division of each PHC Centre, which is based on the total number of consultations provided by the centre. |
| **Peru** |
| The PHC Centre population is by proximity to the PHC Centre, based on areas that the Ministry of Health assigns to each PHC Centre |


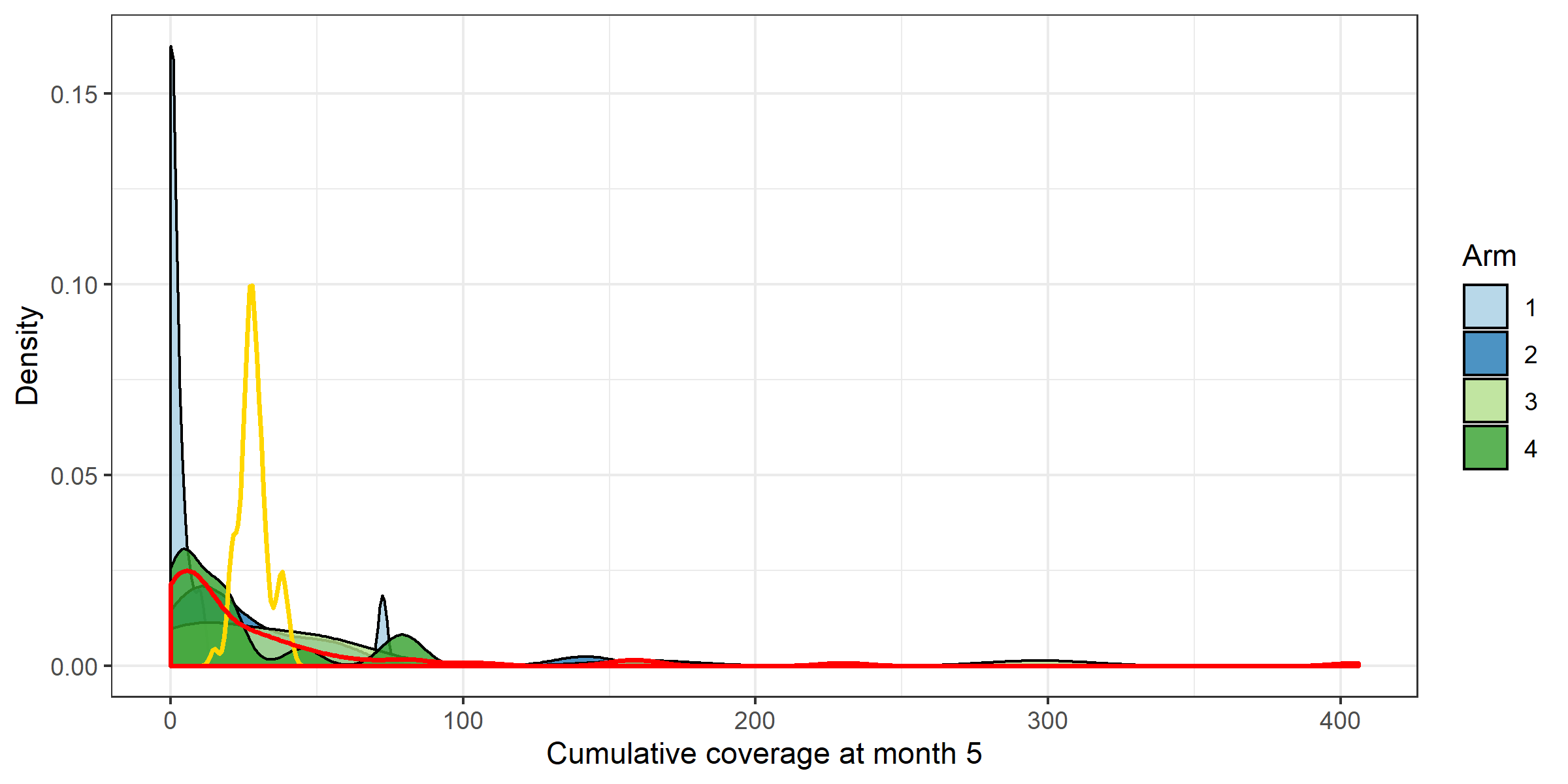


**Appendix Figure 1** Density of cumulative coverage by Arm for month 5. The red and yellow lines indicate hypothetical negative binomial and poisson distributions, respectively, generated from parameters of the empirical distribution. Kolmogorov-Smirnov Tests indicated best fit for negative binomial (D=0.55) relative to poisson distribution (D=0.60) or gamma distribution (D=0.80; not shown in figure due to high peak at low coverage rates, leading to distortion of the scales in the graph).

**Appendix** **Table 2** Primary outcome analyses testing all three hypotheses with inclusion of interaction effects of country and exposure variables. Main effects are constant across countries with the overall effect being comparable to the main models.

| **Appendix Table 2.** Results of additional regression analyses for evaluating Hypotheses 1-3 | | | |
| --- | --- | --- | --- |
|  | Hypothesis 1 | Hypothesis 2 | Hypothesis 3 |
| Exposure ^a^ | -0.551  (-2.671, 1.568) | 3.077**  (0.007, 6.148) | -0.761  (-2.086, 0.564) |
| Country (base: Colombia) |  |  |  |
| Mexico | -1.910*  (-3.827, 0.008) | -0.099  (-2.221, 2.022) | -1.072  (-2.387, 0.242) |
| Peru | -1.911**  (-3.780, -0.042) | -1.488*  (-3.175, 0.199) | -1.393*  (-2.802, 0.016) |
| Baseline coverage rate | 0.055**  (0.013, 0.097) | 0.107*  (-0.010, 0.223) | 0.065***  (0.017, 0.113) |
| Interaction (base: Colombia) | |  |  |
| Exposure ^a^ * Mexico | 0.788  (-1.550, 3.126) | -2.135  (-5.712, 1.442) | 1.294  (-0.297, 2.885) |
| Exposure ^a^ * Peru | 0.468  (-1.806, 2.741) | -0.432  (-3.679, 2.814) | -0.068  (-1.734, 1.597) |
| Intercept | 4.133***  (2.346, 5.919) | 1.055  (-0.473, 2.584) | 3.493***  ^(^2.236, 4.750) |
| Observations | 30 | 29 | 29 |
| Log Likelihood | -80.484 | -69.080 | -87.842 |
| Theta | 2.635^***^ (0.897) | 1.159^**^ (0.584) | 2.228^***^ (0.712) |
| Akaike Inf. Crit. | 174.968 | 152.159 | 189.685 |
| Note: numbers in brackets denote 95% confidence intervals.  ^a^ Exposure variable defined by hypothesis: H1: without (base) vs with municipal support, H2: without (base) vs with training, H3: short (base) vs standard package  *p<0.1; **p<0.05; ***p<0.01 | | | |

## Availability of data

As summarized in ***Appendix Table3***, five months of tally sheet data collection during the implementation period was completed for most PHC Centres. For ten Centres, the full five-month implementation period cycle could not be completed, resulting in possible underestimation of the corresponding coverage rate. As this predominately concerned Arm 3 (4 out of 10 PHCUs with incomplete data, highlighted in ***Appendix Table 3***), the analyses of hypothesis 1 (Arm 2 vs 3) and hypothesis 3 (Arm 3 vs 4) were – if at all –conservatively biased. We compared the proportion of implementation period days without any tally sheet (last column in Appendix Table 3) between Centres who had the full five-month implementation period completed (16.2%) and those that did not (18.1%). According to results from a fractional response regression model, the margin of 1.9% was not significant (coefficient = 0.135, se = 0.709, p = 0.85), indicating negligible impact of missing data in PHCUs that have not completed the 5-months implementation period.

| **Appendix Table 3**. Dates of data collection by Arm | | | | |
| --- | --- | --- | --- | --- |
| **Country** | **Arm** | **Last day of month 5 in implementation period** | **Last day of tally sheet collected** | **Proportion of implementation period days without any tally sheet** |
| Colombia | 1 | 2020-02-15 | No alcohol measurement documented |  |
| Colombia | 1 | 2020-02-15 | 2019-12-02 | 49.3% |
| Colombia | 1 | 2020-04-11 | No alcohol measurement documented |  |
| Colombia | 1 | 2020-04-11 | No alcohol measurement documented |  |
| Colombia | 1 | 2020-02-15 | No alcohol measurement documented |  |
| Colombia | 2 | 2020-02-15 | 2020-02-12 | 2.0% |
| Colombia | 2 | 2020-02-15 | No alcohol measurement documented |  |
| Colombia | 2 | 2020-02-15 | 2020-02-09 | 3.9% |
| Colombia | 2 | 2020-02-15 | 2019-10-25 | 74.3% |
| Colombia | 2 | 2020-04-11 | 2020-03-11 | 20.5% |
| Colombia | 3 | 2020-01-19 | 2019-12-11 | 25.7% |
| Colombia | 3 | 2020-01-19 | 2020-01-16 | 2.0% |
| Colombia | 3 | 2020-01-19 | 2020-01-17 | 1.3% |
| Colombia | 3 | 2020-01-19 | 2020-01-16 | 2.0% |
| Colombia | 3 | 2020-01-19 | 2020-01-16 | 2.0% |
| Colombia | 4 | 2020-01-19 | 2019-12-02 | 31.6% |
| Colombia | 4 | 2020-01-19 | 2019-12-20 | 19.7% |
| Colombia | 4 | 2020-01-19 | No alcohol measurement documented |  |
| Colombia | 4 | 2020-01-19 | 2019-12-20 | 19.7% |
| Colombia | 4 | 2020-03-20 | 2020-03-06 | 9.3% |
| Mexico | 1 | 2020-02-18 | 2020-02-18 | 0.0% |
| Mexico | 1 | 2020-02-02 | 2019-10-22 | 67.8% |
| Mexico | 1 | 2020-03-23 | No alcohol measurement documented |  |
| Mexico | 1 | 2020-02-11 | 2019-10-17 | 77.0% |
| Mexico | 2 | 2020-03-03 | 2020-02-17 | 9.9% |
| Mexico | 2 | 2020-02-18 | 2020-02-18 | 0.0% |
| Mexico | 2 | 2020-02-25 | 2020-02-20 | 3.3% |
| Mexico | 2 | 2020-02-17 | 2020-02-17 | 0.0% |
| Mexico | 2 | 2020-02-24 | 2020-02-11 | 8.6% |
| Mexico | 3 | 2020-03-02 | 2020-02-27 | 2.6% |
| Mexico | 3 | 2020-03-15 | 2020-02-28 | 10.6% |
| Mexico | 3 | 2020-03-01 | 2020-02-21 | 6.0% |
| Mexico | 3 | 2020-04-25 | 2020-01-14 | 67.5% |
| Mexico | 3 | 2020-02-05 | 2020-01-17 | 12.5% |
| Mexico | 4 | 2020-02-23 | 2020-02-21 | 1.3% |
| Mexico | 4 | 2020-02-08 | 2020-02-07 | 0.7% |
| Mexico | 4 | 2020-02-09 | 2020-02-07 | 1.3% |
| Mexico | 4 | 2020-02-29 | 2020-02-20 | 6.0% |
| Peru | 1 | 2020-02-08 | 2020-01-21 | 11.8% |
| Peru | 1 | 2020-02-08 | 2019-10-26 | 69.1% |
| Peru | 1 | 2020-02-08 | No alcohol measurement documented |  |
| Peru | 1 | 2020-02-08 | 2019-12-11 | 38.8% |
| Peru | 1 | 2020-02-08 | No alcohol measurement documented |  |
| Peru | 2 | 2020-02-08 | 2020-01-31 | 5.3% |
| Peru | 2 | 2020-02-08 | 2020-02-08 | 0.0% |
| Peru | 2 | 2020-02-08 | 2020-02-07 | 0.7% |
| Peru | 2 | 2020-02-08 | 2020-02-06 | 1.3% |
| Peru | 2 | 2020-02-08 | 2020-02-05 | 2.0% |
| Peru | 3 | 2020-02-08 | 2020-02-06 | 1.3% |
| Peru | 3 | 2020-02-08 | 2019-10-03 | 84.2% |
| Peru | 3 | 2020-02-08 | 2020-01-15 | 15.8% |
| Peru | 3 | 2020-02-08 | 2020-02-08 | 0.0% |
| Peru | 3 | 2020-02-08 | 2020-02-06 | 1.3% |
| Peru | 4 | 2020-02-08 | 2020-01-15 | 15.8% |
| Peru | 4 | 2020-02-08 | No alcohol measurement documented |  |
| Peru | 4 | 2020-02-08 | 2020-02-07 | 0.7% |
| Peru | 4 | 2020-02-08 | 2020-02-05 | 2.0% |
| Peru | 4 | 2020-02-08 | 2020-02-07 | 0.7% |
| Note. Highlighted are all PHC Centres that had their 5^th^ month of data collection completed after data closure, as determined by lockdown measures in each country. | | | | |

**Appendix Table 4** Description of SCALA municipalities

| **Municipality** | **Intervention or control** | **No of PHCUs in municipality** | **Geographical location in the country** | **Demographic size of municipality** | **Indicators of deprivation** | |
| --- | --- | --- | --- | --- | --- | --- |
|  |  |  |  |  | ***Unemployment rate*** | ***NBI %****** |
| Madrid (COL) | Control | 7 | Municipality in Western Savanna Province and part of the department of Cundinamarca, 21 km outside Bogota. Borders Funza. | 93.154 | 14,9% | 3,2% |
| Funza (COL) | Control | 3 | Municipality in Western Savanna Province and part of the department of Cundinamarca, 25 km outside Bogota. Borders Madrid. | 112.254 | 9,2% | 3,5% |
| Soacha (COL) | Intervention | 10 | Municipality in metropolitan area of Bogota, part of department of Cundinamarca. Capital of Soacha Province, borders Bogota D.C on the east. | 660.179 | 13,8% | 5,3% |
| Miguel Hidalgo (MEX) | Control | 4 | One of 16 municipalities of Mexico City. Located in northwest of Mexico city, west of the historic centre. | 372.889 | 3,0% | no data |
| Xochimilco (MEX) | Control | 3 | One of 16 municipalities of Mexico City. Located in the southeastern part of the city. | 415.007 | 4,0% | no data |
| Álvaro Obregón (MEX) | Intervention | 3 | One of 16 municipalities of Mexico city. Located in southwestern part of the city. | 727.034 | 3,0% | no data |
| Benito Juárez (MEX) | Intervention | 2 | One of 16 municipalities of Mexico city. Located in the north center of Mexico City. | 385.439 | 3,2% | no data |
| Tllapan (MEX) | Intervention/  Control | 6* | One of 16 municipalities of Mexico city. Largest municipality, covering large part of south and southwest area of Mexico city. | 650.567 | 3,9% | no data |
| Chorillos (PER) | Control | 7 | One of the 43 districts of Lima province, located in Lima region. Borders Santiago de Surco in the north and the Pacific ocean in the west. | 314.241 | 6,0%** | 13,1% |
| Santiago de Surco (PER) | Control | 3 | One of the 43 districts of Lima province, located in Lima region. Borders Chorillos in the south. | 329.152 | 6,0%** | 3,6% |
| Callao (PER) | Intervention | 10 | Provincial capital and one of the seven districts in Callao province, part of Callao region. Located at the West area of Lima, and borders the Pacific ocean. | 451.260 | 7,0%** | 11,8% |

*Two of PHCUs are in control arm

**Data only available on regional level (Lima region/Callao region)

*** index to measure multidimnesional poverty, combining information on access to housing, sanitation, education and economic capacity. The NBI methodology seeks to determine whether the basic needs of the population are covered. Groups that do not reach a set minimum threshold are classified as poor. The selected simple indicators are: inadequate housing, housing with critical overcrowding, housing with inadequate services, homes with high economic dependency, homes with school-age children who do not attend school.

**Source material:**

DANE (2018). Censo nacional de población y vivienda. Proyecciones de población. Available from: <https://www.dane.gov.co/index.php/estadisticas-por-tema/demografia-y-poblacion/proyecciones-de-poblacion> [accessed 8.5.2020]

Gobernación de Cundinamarca (2019). Tasa de Desempleo. EM-2017 en Cundinamarca. Available from: <https://mapasyestadisticas-cundinamarca-map.opendata.arcgis.com/datasets/tasa-de-desempleo-em-2017-en-cundinamarca?geometry=-75.261%2C4.520%2C-73.408%2C4.999>) [accessed 8.5.2020]

DANE (2018). Necesidades básicas insatisfechas (NBI). Información Censo nacional de población y vivienda 2018. Available from: <https://www.dane.gov.co/index.php/estadisticas-por-tema/pobreza-y-condiciones-de-vida/necesidades-basicas-insatisfechas-nbi> [accessed 8.5.2020]

INEGI (n.d.). México in cifras: Ciudad de México. Available from: <https://www.inegi.org.mx/app/areasgeograficas/?ag=09#tabMCcollapse-Indicadores> [accessed 8.5.2020]

INEGI (n.d.). Banco de indicadores: Porcentaje de la población de 12 años y más económicamente activa ocupada (Porcentaje), 2015. Available from [https://www.inegi.org.mx/app/indicadores/?t=0070&ag=09014##D00700060](https://www.inegi.org.mx/app/indicadores/?t=0070&ag=09014) [accessed 8.5.2020]

INEI (2017). Censos nacionales 2017: XII Censo de Población, VII de Vivienda y III de Comunidades Indígenas. Sistema de Consulta de Base de Datos. Available from: http://censos2017.inei.gob.pe/redatam/[accessed 8.5.2020]

INEI (n.d.). Sistema de información regional para la toma de decisiones: Tasa de desempleo de la poblacionhttp://systems.inei.gob.pe:8080/SIRTOD/app/consulta [accessed 8.5.2020]

INEI (n.d.). Perú: Mapa de Necesidades Básicas Insatisfechas (NBI), 1993, 2007 y 2017. Available from: <https://www.inei.gob.pe/media/MenuRecursivo/publicaciones_digitales/Est/Lib1588/> [accessed 8.5.2020]

| **Appendix Table 5.** Characteristics of providers by arm | | | | |
| --- | --- | --- | --- | --- |
|  | **Arm 1** | **Arm 2** | **Arm 3** | **Arm 4** |
| **Sample characteristics** | | | | |
| N (PHCUs) | 14 | 15 | 15 | 14 |
| N (all providers) ^1^ | 125 | 201 | 169 | 143 |
| % Female | 78.0% | 75.8% | 77.5% | 66.4% |
| Age (SD) | 38.4 (12.3) | 41.0 (12.6) | 37.7 (12.3) | 37.2 (11.5) |
| Profession (%) |  |  |  |  |
| Doctor ^2^ | 36.0% | 36.8% | 40.2% | 46.8% |
| Nurse | 12.8% | 15.4% | 17.2% | 10.6% |
| Nurse technician | 24.8% | 11.9% | 5.3% | 6.4% |
| Midwife | 3.2% | 11.4% | 5.3% | 8.5% |
| Psychologist | 6.4% | 7.0% | 8.9% | 9.2% |
| Social worker | 8.8% | 5.0% | 4.7% | 4.3% |
| Other | 36.0% | 36.8% | 40.2% | 46.8% |
| Mean number of months participated in study, maximum = 6 (SD) | 5.0 (1.7) | 4.5 (2.1) | 4.5 (2.0) | 4.8 (1.9) |
| Proportion of providers dropped out during study period | 34.4% | 25.4% | 31.4% | 28.7% |
| Note. SD = standard deviation  ^1^ As some providers worked in more than one Arm in Colombia, the number of provider does not add up to the total sample of 622 but to 638.  ^2^ other professions were: | | | | |

**Appendix Table 6** Training coverage by arm and by time of providers joining the project

|  |  | % attending training 1 | % attending training 2 | % attending booster 1 | % attending at least one session |
| --- | --- | --- | --- | --- | --- |
| *Arm 2* | Total | 71.9 |  | 43.3 | 74.1 |
|  | Baseline | 73.0 |  | 43.9 | 79.6 |
|  | M1-M5 | 60.0 |  | 41.2 | 53.7 |
|  |  |  |  |  |  |
| *Arm 3* | Total | 65.4 |  | 49.4 | 66.3 |
|  | Baseline | 61.2 |  | 49.2 | 62.0 |
|  | M1-M5 | 87.5 |  | 50.0 | 83.9 |
|  |  |  |  |  |  |
| *Arm 4* | Total | 67.4 | 59.5 | 50.0 | 76.9 |
|  | Baseline | 67.0 | 58.3 | 51.5 | 75.9 |
|  | M1-M5 | 70.0 | 65.0 | 37.5 | 81.8 |
|  |  |  |  |  |  |
| *Total* | Total | 68.4 | 59.5 | 46.7 | 72.3 |
|  | Baseline | 67.4 | 58.3 | 47.5 | 72.8 |
|  | M1-M5 | 74.6 | 65.0 | 43.5 | 70.2 |

| **Appendix Table 7. Municipal support implemented in the three intervention municipalities until end of implementation month 5.** | | | |
| --- | --- | --- | --- |
| **Municipal action** | **Colombia** | **Mexico** | **Peru** |
| **Local stakeholder groups (CABs)** | Three CAB meetings | Three CAB meetings | Two CAB meetings |
| **Project champion** | One project champion, highly involved in the implementation of programme in the intervention municipality and in the direct communication with Centres. | Two project Champions who participate continuously in the link between health authorities and health providers. Project champion 1 obtained the authorization to use the Mexico City Health Services logo for its incorporation into the project materials. Project champion 2 facilitated the training sessions; encouraged the participation of the suppliers; provided support in the collection of information during the implementation period. | One project champion who provides suggestions for the design and implementation of the SCALA Municipal Action Plan. |
| **Adoption mechanisms** | 1. The benefits of the SCALA project have been emphasized in face-to-face meetings with Centre managers and providers.  2. In implementation month 3, in a face to face meetings with providers, the number of screened patients was communicated.  3. A local university became engaged in the project.  4. In implementation month 3, in a face to face meetings with providers, the highest screening rates per Centre were highlighted.  5. Organizational issues are monitored through discussions with Centres, no substantial issues have been identified. | 1. During the training sessions, the benefits of implementing the screening and brief advice in the Centre for patients, providers and the community have been highlighted.  2. In the training sessions, the large number of patients that can benefit if screening and brief advice are implemented in the Centre was reaffirmed.  3. A poster presentation held at an Annual Research Meeting of the National Institute of Psychiatry; a presentation about the role of alcohol screening was held on the National Day against Harmful use of Alcoholic Beverages 2019.  4. Informing Centres about the percentage of screenings carried out by each Centre is done on a monthly basis.  5. Organizational issues are monitored through discussions with Centres, no substantial issues have been identified. | **1.**  There has been work with the Mental Health Program of the Ministry of Health, in order to promote the adoption of the program in the implementation municipality.  **2.** The large number of patients who benefit from the project is communicated to providers, focusing on three subgroups with higher alcohol risk in the intervention municipality: a) persons in treatment of tuberculosis, b) persons at risk of sexual transmitted diseases, c) persons in violent families.  **3.** In order to engage the municipality, 35 community promoters have been trained in methods for working in alcohol prevention.  **4.** Lists were created for each Centre using WhatsApp to promote the identification of champions. **5.** Organizational issues are monitored through discussions with Centres; one issue identified is that providers seem very busy. |
| **Support systems** | **1.** Training packages were slightly shortened, in order to fit into the Centres’ schedules and rules of attendance of providers.  **2.** One formal meeting was organized in the first two months of implementation to identify difficulties regarding the brief intervention and the care pathway. It was identified that providers still needed support to get used to the exact pathway and, therefore, three short support videos were created, about how to fill in the tally sheets, how to mark the boxes and what is the needed material to be delivered for each case. **3.** Meetings for feedback with providers are held every two months, in which the screening rates are communicated.  **4.** Not yet implemented  **5**. Not yet implemented | **1.** Materials and activities of the training sessions (i.e. role playing, presentations and analysis of the videos) were adjusted to the needs of each Centre. **2.** No additional tailoring was needed. **3.** Reporting each month to Centres the number of screenings; informing every three months Centres on the progress of the global project  **4.** Not yet implemented  **5**. Not yet implemented | **1.** Additional materials have been added for new providers who did not have previous information about the program. **2.** No additional tailoring needed. **3.** Reporting each month to Centres the number of screenings.  **4.** Not yet implemented **5.** Exploring the option of involving Community Mental Health Services, who could train other Centres in the future. |
| **Communication campaign** | A total of ~50 posters have been placed in the participating Centre’s and in public places such as cafeterias, drugstores and small stores.  Additionally, monthly WhatsApp messages regarding the project and/or importance of alcohol screening are sent to providers. | A total of ~800 posters have been placed in 8 of the 9 participating PHCUs in the intervention municipality and in other public places such as: grocery stores, stationers shops, restaurants, parks and public markets. Additionally, ~500 pocket calendars for providers in Centres, ~250 pocket calendars for patients, ~350 desk calendars, ~350 pocket calendars and ~40 pin buttons have been distributed in Centres, to providers and patients. | A total of ~800 posters have been placed in the participating Centres of the intervention municipalities and in other public places, such as markets, universities, bus-stops.  Three promotional videos have been displayed in participating Centres.  Additionally, monthly WhatsApp messages regarding the project and/or importance of alcohol screening are sent to providers. |
